# Pattern and control of biomass allocation across global forest ecosystems

**DOI:** 10.1002/ece3.3089

**Published:** 2017-06-14

**Authors:** Yongtao Jiang, Limei Wang

**Affiliations:** ^1^ College of Environmental Science and Traveling Nanyang Normal University Nanyang Henan China; ^2^ Provincial Key Laboratory of Remote Sensing Monitoring College of Environmental Science and Traveling Nanyang Normal University Nanyang Henan China

**Keywords:** allometric model, belowground biomass, biomass allocation, forest ecosystems, global environmental change, *root:shoot ratios*

## Abstract

The underground part of a tree is an important carbon sink in forest ecosystems. Understanding biomass allocation between the below‐ and aboveground parts (*root:shoot ratios*) is necessary for estimation of the underground biomass and carbon pool. Nevertheless, large‐scale biomass allocation patterns and their control mechanisms are not well identified. In this study, a large database of global forests at the community level was compiled to investigate the *root:shoot ratios* and their responses to environmental factors. The results indicated that both the aboveground biomass (*AGB*) and belowground biomass (*BGB*) of the forests in China (medians 73.0 Mg/ha and 17.0 Mg/ha, respectively) were lower than those worldwide (medians 120.3 Mg/ha and 27.7 Mg/ha, respectively). The *root:shoot ratios* of the forests in China (median = 0.23), however, were not significantly different from other forests worldwide (median = 0.24). In general, the allocation of biomass between the belowground and aboveground parts was determined mainly by the inherent allometry of the plant but also by environmental factors. In this study, most correlations between *root:shoot ratios* and environmental factors (development parameter, climate, altitude, and soil) were weak but significant (*p *<* *.01). The allometric model agreed with the trends observed in this study and effectively estimated *BGB* based on *AGB* across the entire database.

## 1 INTRODUCTION

The forest ecosystem holds forty percent of the global belowground carbon. It plays key roles in climate change and carbon cycling (Dixon et al., 1994). New biomass produced by photosynthesis is transported to the leaves, stems, roots, and reproductive organs. When new biomass is proportionally allocated to these sinks, it also ensures proportional distribution of new carbon (Reich et al., 2014). Biomass allocation is influenced by both biotic and abiotic factors. Understanding biomass distribution is essential for global carbon cycle modeling and accounting (Hui, Wang, Le, Shen, & Ren, 2012). The *root:shoot ratios* effectively describe the allocation between aboveground biomass (*AGB*) and belowground biomass (*BGB*). They can therefore be used to estimate BGB using the readily measurable AGB (Wang et al., 2014). The *root:shoot ratios* reflect the adaptation of a plant to various environments (Mokany, Raison, & Prokushkin, 2006). This information is a necessary input of carbon modeling. Changes in *BGB* and C content may be predicted when the driving forces of biomass allocation are identified.

Over the past twenty years, many scholars have used allometry to study biomass allocation in plants (Enquist & Niklas, 2002; West, Brown, & Enquist, 1997; West, Enquist, & Brown, 2009). Their hypotheses have been corroborated by experimental data (Niklas, 2006; Yang, Fang, Ji, & Han, 2009). Biomass partitioning is described using the allometric model *BGB* = a*AGB* ^b^, where a is a normalizing scaling constant, and b is an allometric scaling exponent (Huxley & Tessier, 1936; Niklas & Enquist, 2001). *BGB* scales nearly isometrically with respect to *AGB* for both woody and nonwoody plants (Enquist & Niklas, 2001, 2002). This model has been validated across ecologically diverse species worldwide using plants with a wide range of total body mass at the individual plant level. (Niklas, 2005, 2006; Niklas & Enquist, 2002). Similarly, many allometric *BGB–AGB* models have also been proposed for the major forest types in China (Cheng & Niklas, 2007; Hui et al., 2014; Luo, Wang, Zhang, Booth, & Lu, 2012). These were based on community‐level data sets. Some studies indicated that the scaling exponents between *AGB* and *BGB* varied by forest origin, phylogeny, leaf habit, forest type, stand age, and climate (Hui et al., 2014; Luo et al., 2012) on different scales. Others reported that using different regression methods might result in scaling exponent differences (Li, Han, & Wu, 2005). Although allometric models may be useful for predicting *BGB* at specific sites and in particular species (Brown, 2002; Li, Kurz, Apps, & Beukema, 2003; Mokany et al., 2006), more evidence is required to demonstrate a universal scaling relationship. Once this parameter is fully validated, allometric relationships can be applied to predict *BGB* across wide temporal and spatial scales.

Many studies have indicated that various factors influence *AGB–BGB* allocation. These include species characteristics, stand development, stand density, resources, and climate (Cairns, Brown, Helmer, & Baumgardner, 1997; Cambui et al., 2011; Poorter & Nagel, 2000). Plants adjust their biomass allocation between the belowground and aboveground parts (*root:shoot ratios*) in ways characteristic of each species (Monk, 1966). Temperature, precipitation, solar radiation, soil texture, and resource availability (such as soil moisture and nitrogen) significantly affect biomass allocation (Mokany et al., 2006; Reich, 2002). In general, environmental stressors force plants to invest their resources in organ growth (Cairns et al., 1997; Cambui et al., 2011; Poorter & Nagel, 2000). Previous studies have investigated variations in the *root:shoot ratios* associated with species characteristics, stand development, stand density, resources, soil texture, and climate at the regional or global level (Cairns et al., 1997; Cambui et al., 2011; Mokany et al., 2006; Poorter & Nagel, 2000; Reich, 2002; Zhang, Song, et al., 2015; Zhang, Wang, et al., 2015), but they reported different findings. More evidence is needed to determine whether the *root:shoot ratios* responds on a large scale to biotic and abiotic factors. Several studies have investigated the forests of China and cited significant findings (Hui et al., 2014; Luo, Wang, Zhang, Ren, & Poorter, 2013; Luo et al., 2012; Wang, Fang, & Zhu, 2008; Zhang, Song, et al., 2015; Zhang, Wang, et al., 2015), but these results may not be consistent with those of the forests elsewhere.

The mechanisms by which plants partition photosynthate under environmental stress are incompletely understood. In the short term, the responses of the *root:shoot ratios* to biotic and abiotic factors are predictable for individual species (Chapin, 1980; Hawkins, Kiiskila, & Henry, 1999; Mooney et al., 1988; Vogel et al., 2008). Nevertheless, it is difficult to forecast large‐scale biomass allocation patterns in response to biotic and abiotic stressors, and on a global scale, the results are inconsistent. In the effort to identify large‐scale biomass allocation patterns across forest ecosystems, data were gathered from the literature worldwide for *root:shoot ratios*,* BGB,* and *AGB* or for standing leaf, stem, flower and fruit dry weights, respectively. Geographical factors, vegetation parameters, and environmental variables were also determined to evaluate biomass allocation in response to biotic and abiotic factors. Data were specifically collected for the forests of China. The following questions were addressed in this study: (1) on a global scale, how do trees partition their biomass between organs and between aboveground and belowground parts? (2) how does the allometric theory hold up at the individual plant and community levels? (3) how do biotic and abiotic variables affect the *root:shoot ratios*? and (4) on a worldwide basis, how well does the allometric theory predict *BGB* (root biomass)?

## 2 MATERIALS AND METHODS

### 2.1 Data collections

All correlations between the *root:shoot ratios* and biotic and abiotic factors were analyzed using the data sets of Luo et al. (2012) and Mokany et al. (2006). Luo et al. (2012) reported 1,138 pairs of *AGB* and *BGB* measurements for about 250 types of forest at 343 sites across China. They compiled this data set from 511 sources published between 1978 and 2008. In this study, 415 pairs of *AGB* and *BGB* measurements were gathered from the published literature. These were added to the Luo et al. (2012) data set. Therefore, in this study, a total of 1,553 pairs of *AGB* and *BGB* measurements for the forests of China were collected. The Mokany et al. (2006) data set was compiled for forests worldwide and consisted of 786 pairs of *AGB* and *BGB* measurements obtained from 266 sources (books, published reports, and conference reports). Only studies presenting pairs of data for both *AGB* and *BGB* were included in Mokany et al. (2006) data set. Biomass data for individual plants and *BGB* derived from models were excluded.

In the aforementioned data sets, the following factors (where available) were recorded for each sampling site: biomass (Mg/ha); *AGB* (or leaf, stem, branch, flower, and fruit biomass if available); *BGB*; site description (location, longitude, latitude, elevation, soil texture, and soil nutrient profile); climate (mean annual precipitation (*MAP*), mean annual temperature (*MAT*), mean annual evapotranspiration (*ET*), mean annual potential evapotranspiration (*PET*), and sunshine duration (*SH*)); vegetation characteristics (forest origin, forest type, and dominant species); and stand parameters (stand age, mean tree height, stem density, and mean diameter at breast height [*DBH*]). *ET* and *PET* were derived from 1 km^2^ land surface *ET* data sets reported by the Numerical Terra Dynamic Simulation Group at http://www.ntsg.umt.edu/project/mod16.

In the Luo et al. (2012) data set, missing *MAT*,* MAP* and *SH* data for each site were estimated by minimum‐distance interpolation using 664 ground observation stations across China. The mean *MAT*,* MAP*, and *SH* for 1961–2010 were used in the analysis. Estimates of *MAT* and *MAP* were compared with measured values from the literature. The estimated *MAP* was significantly correlated with the measured *MAP* ($R^{2}=.92,\;p<.01$). The estimated *MAT* was also significantly correlated with measured *MAT* ($R^{2}=.90,\;p<.01$). Soil texture data for the forests of China were obtained from texture maps (Institute of Soil Science, Chinese Academy of Sciences, 1986) and were based on the location (longitude and latitude) of the data site. For the Mokany et al. (2006) data set, missing *MAT* and *MAP* were estimated using a Local Climate Estimator (*LocClim*, FAO, 2002). This tool estimates basic climate variables from the latitude, longitude, and altitude. The accuracies of the estimated *MAT* and *MAP* were validated by comparing them with the literature measurements (Mokany et al., 2006).

The generality of the allometric partitioning theory was tested using two community‐level data sets (i.e., Luo et al. (2012) and Mokany et al. (2006)) and one data set at the individual plant level(Enquist and Niklas (2002)). The latter data set included both woody and nonwoody plants. For woody plants, 346 biomass records were selected from Edwards (1983). Data for nonwoody plants in Enquist and Niklas (2002) were collected from primary literature published between 1987 and 2002. All standing biomass units were converted into kilogram of dry weight per plant. Enquist and Niklas (2002) cited 257 woody and nonwoody species, and more than one thousand dry mass measurements spanning ten orders of magnitude of *AGB* and *BGB*. Only biomass data (leaf, stem, and root biomass in kg dry matter/plant) were presented in the Enquist and Niklas (2002) data set. Therefore, it was only used to analyze allometric relationships in plant organ biomass.

### 2.2 Statistical analyses

Linear regression analysis was used to evaluate the relationship between the *root:shoot ratios* and biotic and abiotic factors. Ordinary least squares (*OLS*) were used to establish regression formulas to forecast *BGB* based on *AGB* measurements. The reliability of the *BGB* predicted from *AGB* measurements was determined by calculating the % prediction errors $\text{Pred.Error}=\left[\left(\text{Obs.}BGB-\text{Pred.}BGB\right)/\text{Pred.}BGB\right]\times 100$ (Smith, 1980).

For woody plants, allometric models predict that standing leaf biomass ($M_{L}$) scale as the 3/4 power of both the standing stem biomass ($M_{S}$) and the standing *BGB* ($M_{R}$), and that the $M_{R}$ scale is indirectly proportional to $M_{S}$ (Enquist & Niklas, 2002; Niklas, 2005). Assuming that the roots are the only underground organs, $M_{R}=M_{B}$, then, $M_{L}=\beta M_{S}^{3/4}=\beta M_{R}^{3/4}$, $M_{S}={\left(\beta_{1}/\beta \right)}^{4/3}M_{R}$ and $\beta_{1}M_{B}^{3/4}+{\left(\frac{\beta_{1}}{\beta }\right)}^{4/3}M_{B}=M_{A}$.

The regression curve describing the allometric relationship had the form $logM_{1}=log\beta +\alpha logM_{2}$, where $M_{1}$ and $M_{2}$ are the plant organ biomasses, logβ is the y‐intercept of the regression curve (the allometric constant in *RMA* analyses), and α is the slope of the regression curve (the allometric scaling exponent in *RMA* analyses). Reduced major axis (*RMA*) regression was applied to establish allometric models for log‐transformed data for *BGB* and *AGB* (Enquist & Niklas, 2002; Niklas, 2005). The significance of the differences between slopes (allometric scaling exponent) of the *RMA* regression formulas was evaluated using the univariate analysis of variance function in SPSS V. 17.0.

## 3 RESULTS

### 3.1 Variations in *AGB, BGB*, and *root:shoot* ratios

**Figure 1:**
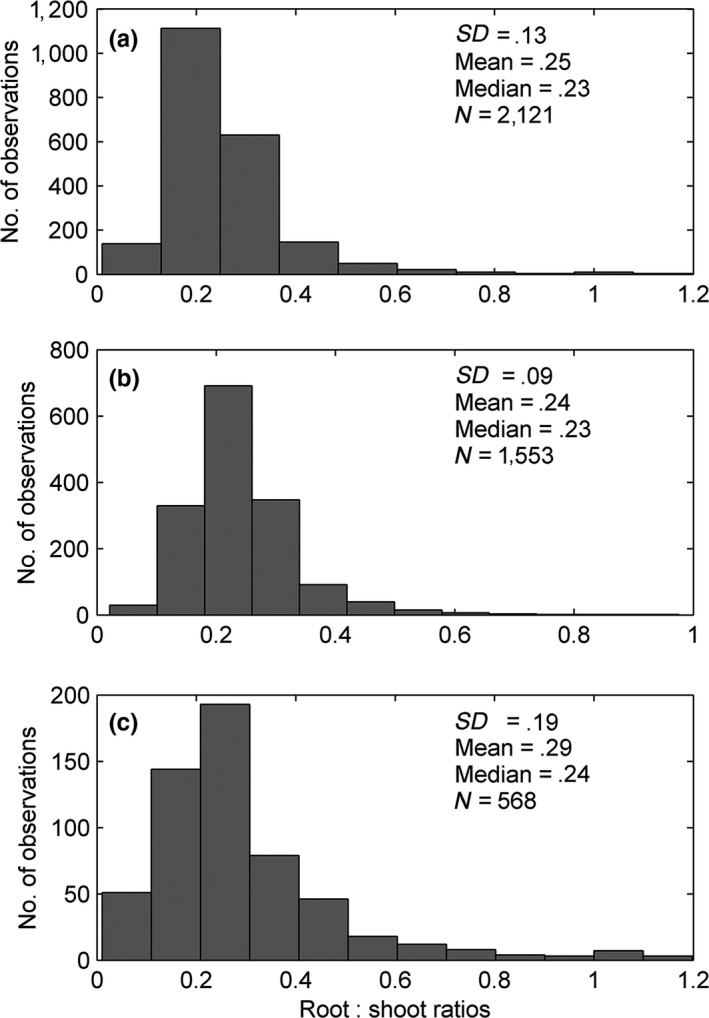
Distribution of *root:shoot ratios* for (a) all forests including those in China (b) Chinese forests (c) global forests. Mean and Median, the mean and median values of the *root:shoot ratios,* respectively; SD, the standard deviation; N, number of observations. Summary of statistics provided in Table 1

Both *AGB* and *BGB* varied significantly in the forests of China and worldwide. *AGB* ranged from 0.054 to 1,433 Mg/ha in the forests of China and from 0.058 to 1,736 Mg/ha worldwide. *BGB* ranged from 0.0089 Mg/ha in Chinese forests and from 0.046 to 204 Mg/ha in global forests. Table 1 shows that the standing biomass in the forests of China is significantly lower than that of global forests. The *root:shoot ratios* varied significantly in the forests of China (0.02–0.98) and the rest of the world (0.01–1.20). Nevertheless, *root:shoot ratios* did not significantly differ between the forests of China and those elsewhere; their mean and median values were similar (Figure 1).

**Table 1:** Comparison of *AGB*,* BGB* and the *root:shoot ratios* in global forests and those in China

Forest group	*AGB* (Mg ha^−1^)				*BGB* (Mg ha^−1^)				*Root:shoot ratios*			
	Mean	Median	SD	*n*	Mean	Median	SD	*n*	Mean	Median	SD	*n*
China's forests	96.78	73.04	85.87	2,010	22.27	17.03	18.34	1,540	0.24	0.23	0.09	1,553
Global forests	161.27	120.34	167.57	566	35.69	27.65	34.16	568	0.29	0.24	0.19	568
Total	110.95	79.35	112.37	2,576	25.88	18.45	24.39	2,107	0.25	0.23	0.13	2,121

*AGB*, aboveground biomass; *BGB*, belowground biomass; SD, standard deviation; *n*, number of observations.

### 3.2 Factors influencing forest biomass and *root:shoot* ratios

**Figure 2:**
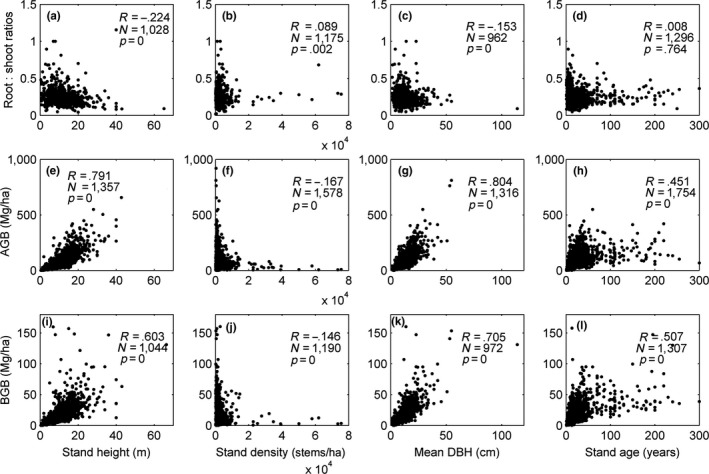
Variations in *root:shoot ratios* (a–d), *AGB* (e–h), and *BGB* (i–l) with tree height, stand density, mean diameter at breast height (*DBH*), and stand age. R, correlation coefficient; p, statistical significance; N, number of observations

Both *AGB* and *BGB* increased significantly (*p *<* *.01) with stand height, mean *DBH*, and stand age. On the other hand, both *AGB* and *BGB* were negatively correlated with stand density (*p *<* *.01). The *root:shoot ratios* decreased significantly (*p *<* *.01) with increasing stand height and mean *DBH*, and increased slightly with stand density (Figure 2). There was no significant relationship between *root:shoot ratios* and stand age.

**Figure 3:**
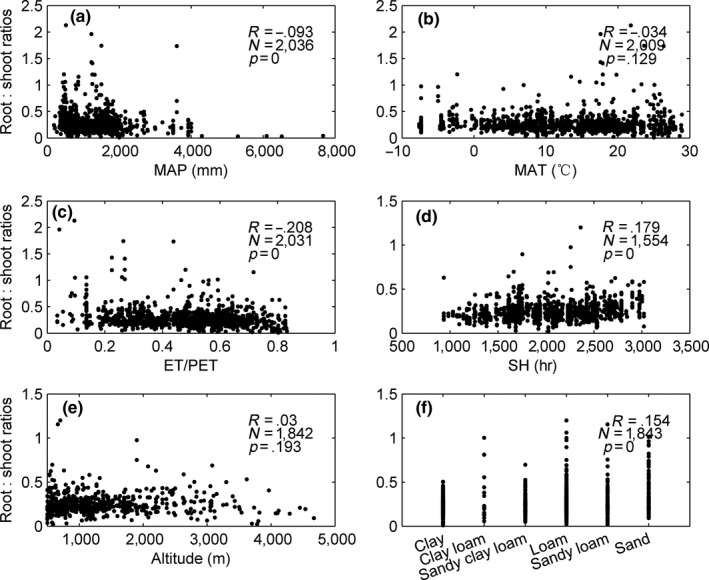
*Root:shoot ratios* as a function of (a) *MAP*, (b) *MAT,* (c) *ET/PET,* (d) *SH,* and (e) Altitude. (f) Soil texture. R, correlation coefficient; *p*, statistical significance; n, number of observations

*The root:shoot ratios* decreased significantly (*p *<* *.01) with increasing *MAP* and *ET/PET* (Figure 3a–c). In contrast, the *root:shoot ratios* increased significantly (*p *<* *.01) with *SH*. Nevertheless, neither *MAT* nor altitude significantly affected the *root:shoot ratios* (Figure 3d–e).

The *root:shoot ratios* in clay and loam soils were significantly lower than those in sandy and sandy loam soils (Figure 3f). Clay loam soil, however, had significantly higher *root:shoot ratios* than those of any other soil texture.

Overall, climate factors (MAP, MAT, SH, and ET/PET) and soil texture explained 34 percent of the variability in *root:shoot ratio* data, and all relationships were significant (*p *<* *.01) except for MAP (Table 2).

**Table 2:** Results of the analysis of covariance using the *root:shoot ratios* as the dependent variable, the mean annual precipitation (*MAP*), mean annual temperature (*MAT*), sunshine hours (*SH*), and *ET/PET* as covariates, and the soil texture as the factor. df, degrees of freedom; MS, mean square

Source	df	MS	*F*‐ratio	*P* value
Soil texture	5	168.90	18.56	<.0001
MAP	1	9.20	1.01	.315
MAT	1	248.97	27.36	.0001
SH	1	107.85	11.85	.001
ET/PET	1	883.33	97.06	.0001
Residual	2,046	9.10		

### 3.3 Allometric models

**Figure 4:**
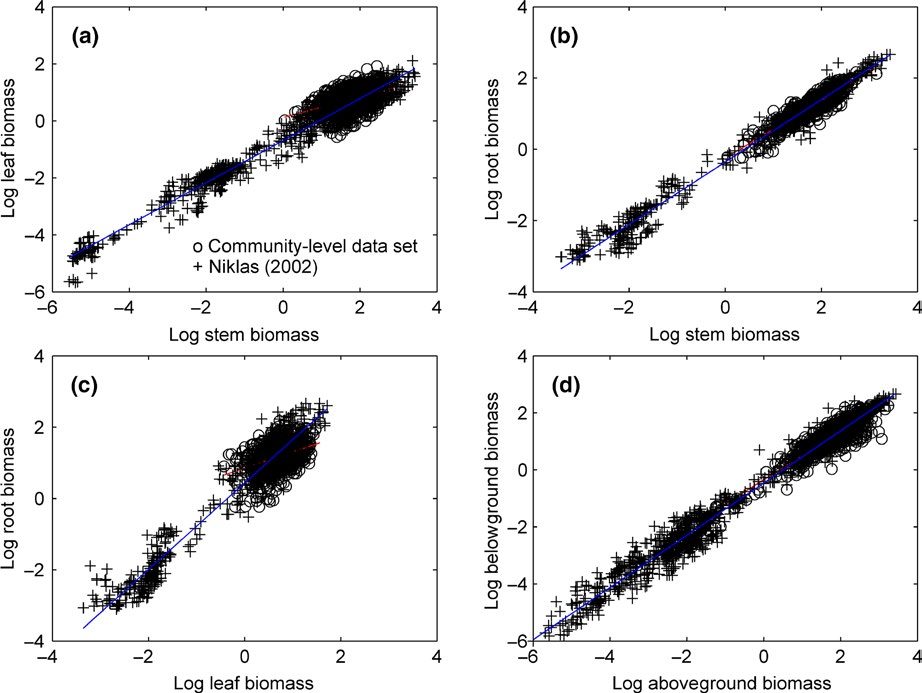
Allometric plots of log‐transformed data for (a) leaf and stem biomass(Sig. *p* = 0), (b) root and stem biomass (Sig. *p* = 0), (c) root and leaf biomass (Sig. *p* = 0), and (d) below‐ and aboveground biomass (Sig. *p* = 0). Solid lines represent *OLS* regression curves for the Niklas (2005) and Niklas and Enquist (2002) and community‐level data sets. All correlations are significant at *p *<* *.01. Sig. *p* indicates the significance of difference in the slopes of the linear regression between two data sets. Sig. *p* > .05 means no significant difference in regression slopes; Sig. *p* < .05 means significant difference in regression slopes

The scaling exponent of the allometric model for $M_{B}$ and $M_{A}$ ranged from 0.75 to 1.00 for woody plants (Enquist & Niklas, 2001; Niklas, 2005). These predictions ($\alpha_{Pred.}$ in Table 3) aligned with the trends in the entire database (Table 3). In general, data presented at the individual plant level (kg dry matter/plant)—fit best with the allometric model (Figure 4; Table 3).

**Table 3:** RMA analysis of log‐transformed data for organ biomass

	$\alpha_{Pred.}$	$\alpha_{RMA}\pm SD$	95% CI	$log\beta_{RMA}$ ±SD	95% CI	*n*	r^2^
Across entire database							
$M_{B}$ versus $M_{A}$	3/4‐1	0.920 ± 0.01	0.915–0.924	−0.479 ± 0.01	−0.492 to −0.466	3,109	.980
$M_{B}$ versus $M_{S}$	1.0	0.882 ± 0.00	0.875–0.889	−0.375 ± 0.01	−0.388 to −0.362	1,616	.974
$M_{B}$ versus $M_{L}$	4/3	1.189 ± 0.01	1.169–1.209	0.323 ± 0.01	0.302–0.344	1,621	.882
$M_{L}$ versus $M_{S}$	3/4	0.775 ± 0.01	0.766–0.783	−0.653 ± 0.01	−0.662 to −0.643	2,546	.927
Across community‐level database							
$M_{B}$ versus $M_{A}$	3/4‐1	0.938 ± 0.01	0.918–0.957	−0.521 ± 0.01	−0.557 to −0.484	2,105	.811
$M_{B}$ versus $M_{S}$	1.0	0.887 ± 0.01	0.865–0.909	−0.392 ± 0.02	−0.433 to −0.351	1,101	.817
$M_{B}$ versus $M_{L}$	4/3	1.088 ± 0.03	1.029–1.146	0.355 ± 0.03	0.305 to 0.404	1,101	.166
$M_{L}$ versus $M_{S}$	3/4	0.867 ± 0.02	0.827–0.906	−0.783 ± 0.04	−0.854 to −0.712	1,549	.176

All cases were significant (*p* < .01).

$M_{A}$, aboveground biomass; $M_{B}$, belowground or root biomass; $M_{S}$, stem biomass; $M_{L}$, leaf biomass; *SD*, standard deviation; *n*, number of observations; $\alpha_{Pred.}$, model‐predicted scaling components constructed by Niklas (2005) and Niklas and Enquist (2001, 2002).

Although there were significant differences (Sig. *p* = 0) in the allometric slopes for $M_{L}$ versus $M_{S}$, $M_{R}$ versus $M_{S}$, and $M_{R}$ versus $M_{L}$ (Figure 4a–c), no significant differences (Sig. *p* = .05) were found for $M_{B}$ versus $M_{A}$ (Figure 4d) between the individual plant and community‐level data sets (Figure 4).

## 4 DISCUSSION

### 4.1 Variations in *AGB, BGB*, and *root:shoot* ratios

Both biotic and abiotic factors (species, stand development, origin, human activities, resources, and climate) accounted for wide variations in *AGB, BGB*, and *root:shoot ratios* in both global and Chinese forests. Table 1 shows that both AGB and BGB of the forests of China were much lower than those of global forests. Local studies indicated that the biomass of typical forests in China is roughly on the same level as that of the rest of the world (Fang, Liu, & Xu, 1996; Peng & Zhang, 1994). Most original zonal forests of China are replaced by secondary forests with lower biomass. Moreover, in China, the tropical forests of China are situated near the northern edge of the tropical zone, and the temperate forests are drought‐prone. These environmental disadvantages may explain the fact that the forests of China have lower *AGB* and *BGB* than typical global tropical and temperate forests (Fang et al., 1996).

### 4.2 Factors influencing forest biomass and *root:shoot* ratios

The response of *root:shoot* to various biotic and abiotic factors was examined. The variables included stand height, stand density, mean *DBH*, stand age, precipitation, temperature, *ET*,* SH*, altitude, and soil type. Previous reports have indicated that *root:shoot ratios* are either positively or negatively correlated with annual precipitation at the local or regional scale (Kang et al., 2013; Li et al., 2012; Wang et al., 2008) but decreased with increasing precipitation on the global scale (Mokany et al., 2006). Temperature also influenced the *root:shoot ratios* differently with region and vegetation type (Luo et al., 2013; Read & Morgan, 1996). Soil texture, nutrient availability, vegetation type, and plant structure also influenced the *root:shoot ratios* across ecosystems and regions (Mokany et al., 2006). Data sets based on specific regions and precise classifications may account for general tendencies in *root:shoot ratio* variations and explain the regional differences in the responses of the *root:shoot ratios* to the same factor.

A key contribution of this study is the investigation of the response of *root:shoot* to biotic and abiotic factors using a large worldwide database. The *root:shoot ratios* were negatively correlated with *MAP* and *MAT* and positively correlated with *SH* (Figure 3). A comprehensive *ET*/*PET* index may best describe the response of the *root:shoot ratios* to climate. The *root:shoot ratios* also increased slightly with altitude. This finding is consistent with previous studies (Leuschner, Moser, Bertsch, Roderstein, & Hertel, 2007; Luo et al., 2005). Changes in climate with altitude may cause variations in the *root:shoot ratios*.

In general, the *root:shoot ratios* increased with forest soil coarseness (from clay to sand). The *root:shoot ratios* were significantly higher in sand and sandy loam soils than those in clay and loam soils. The relatively lower water content and nutrient availability of coarse soil may explain the large *root:shoot ratios*. It is easier to sample root biomass from sandy than finer soils, and this property may also account for the higher *root:shoot ratios* in coarser soils (Mokany et al., 2006).

Although both *AGB* and *BGB* had high positive correlations with stand height, mean *DBH*, and stand age, these factors were only weakly (but significantly) correlated with the *root:shoot ratios* (Figure 2). Therefore, the *root:shoot ratios* may be genetically stable despite multiple environmental stressors.

Young plantation stands (<10 years) were included in all analyses although they are affected by traditional practices such as selected harvest and thinning which may influence the *root:shoot ratios*(King et al., 2007; Sheng and Fan, 2005; Luo et al., 2012). The lack of field observations for these forests, however, prohibited further analysis in this study. Fortunately, there were very few young plantations included in the analysis, and so the effects of selected harvest and thinning on the *root:shoot ratios* can be safely ignored here.

Plants tend to adapt to environment variations by adjusting their *root:shoot ratios* (Friedlingstein, Joel, Field, & Fung, 1999; McConnaughay & Coleman, 1999). The analyses of this study indicated that biomass allocation between the belowground and aboveground parts is determined mainly by the inherent allometric relationships in the plant but also environmental factors. Most of the correlations between the *root:shoot ratios* and environmental parameters were significant (*p *<* *.01) but weak. Biomass allocation has been changing for thousands of years, whereas documented measurements of biomass, climate, and soil properties span only several decades. Most of the earlier studies on environment allocation correlations were based on data from sporadic experiments. One or two factors were studied, whereas the others were fixed or suppressed (Agren & Franklin, 2003; Gholz et al., 1991; Kellomaki & Wang, 1996; Li et al., 2012; Matsui, Fukuda, Inoue, & Matsushita, 2003; Read & Morgan, 1996). Nevertheless, biomass allocation is the result of complex environmental factors that short‐term studies cannot represent. For this reason, despite decades of research, no strong or general correlations were found between biomass allocation and environment factors at the global scale.

### 4.3 Allometric models

The regression slopes across the entire database at the individual plant level were predicted by models ($\alpha_{Pred.}$; Niklas, 2005; Niklas & Enquist, 2001, 2002) and used in comparisons across the data sets from this study. RMA regression analysis showed that the slope ($\alpha_{\mathrm{RMA}}$ = 0.938) of the regression curve $M_{B}$ versus $M_{A}$ for the community‐level data set is not significantly different (*p *>* *.01) from that predicted by the allometric model ($\alpha_{\mathrm{Pred}.}$ = 3/4‐1). This finding confirmed the hypothesis that $M_{B}$ versus $M_{A}$ relationships at the community and individual plant levels share the same scaling exponents (*RMA* regression curve slopes). Niklas (2005); Niklas and Enquist (2002) constructed their allometric model based on a wide range of data for woody and nonwoody plants worldwide. When the nonwoody species data were excluded, the relationships observed for $M_{B}$ versus $M_{A}$ were even more consistent with the allometric model prediction. Unfortunately, the Enquist and Niklas (2002) data set did not present a definitive standard distinguishing woody from nonwoody species. When it was combined with the Enquist and Niklas (2002) data set, the related regression curve coefficients improved (Table 3).

**Figure 5:**
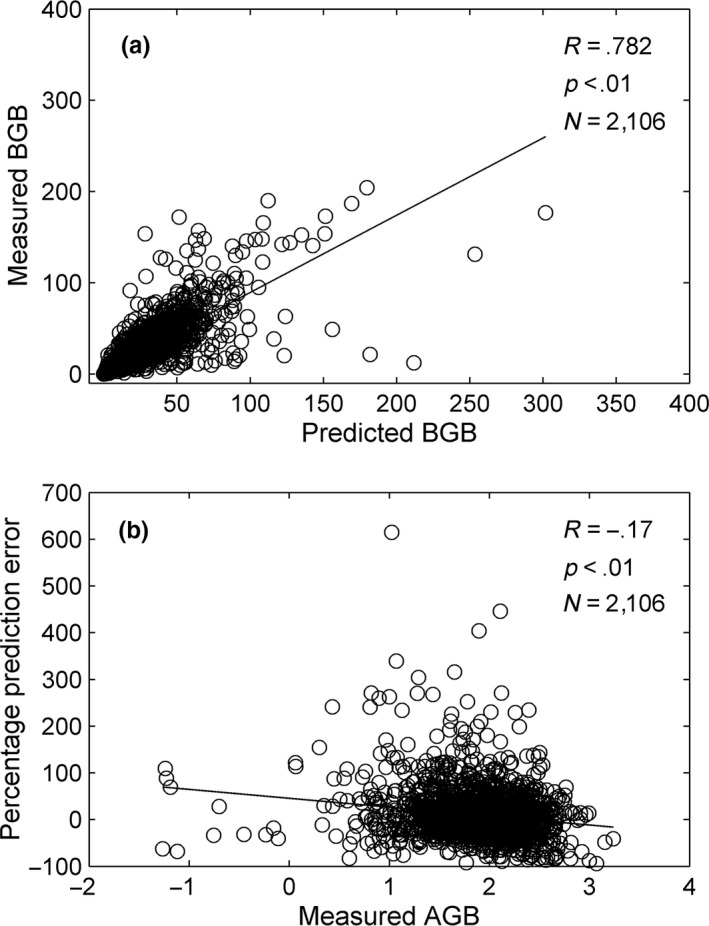
Accuracy of *OLS* regression formulas in predicting *BGB* based on *AGB*. (a) correlation analysis between measured and predicted *BGB*, and (b) variation in percentage prediction error with *AGB*

It is apparent that the allometric theory is completely empirical. The slopes and regression curve constants vary with region and vegetation type. Various data sets yield different slope and constants (Cheng & Niklas, 2007; Luo et al., 2012; Mokany et al., 2006; Yang et al., 2009). This study predicted the community‐level *BGB* from the *AGB* using the $M_{B}$ versus $M_{A}$ *OLS* regression formula across the entire database. It was found that the allometric relationship reasonably estimates *BGB* (Figure 5a). The percentage error of the *BGB* predicted by *AGB* using *OLS* regression formula decreased as *AGB* increased (Figure 5b). That is, the reliability of the *OLS* regression formula increased with *AGB* (or plant size) across global forest communities. The precise estimation of allocation patterns is essential for predicting global carbon budget and climate change, and for ecosystem modeling. Although many carbon allocation schemes were constructed in the last few decades, none of them accurately described the long‐term allocation dynamics in various environments. The allometric theory empirically evaluates global root biomass but its scaling components vary with environmental conditions. It also indicates that it will be possible to trace biomass allocation and determine when it reaches homeostasis.

## 5 CONCLUSION

Using a large database of global forest ecosystems, the *root:shoot ratios* and their responses to environmental factors were investigated in this study. Both aboveground and belowground biomass in the forests of China were lower than those of global forests. Nevertheless, the *root:shoot ratios* were not significantly different from each other. They were determined primarily from the inherent allometric relationships of plants, but they were significantly affected by developmental parameters, climate variables, altitude, and soil (*p *<* *.01).

*The root:shoot ratios* responded to changes in mean annual temperature, mean annual precipitation, and the potential water deficit index. They were negatively correlated with mean annual precipitation, mean annual temperature, and potential water deficit. Soil texture, developmental parameters, and climatic conditions influenced the magnitudes of the *root:shoot ratios*. The allometric theory aligned with the trends observed in this study and correctly estimated *BGB* based on *AGB* for the entire database.

## CONFLICT OF INTEREST

None declared.

## References

[ece33089-bib-0001] Agren, G. I. , & Franklin, O. (2003). Root: shoot ratios, optimization and nitrogen productivity. Annals of Botany, 92, 795–800. 14565938 10.1093/aob/mcg203 PMC4243620

[ece33089-bib-0002] Brown, S. (2002). Measuring carbon in forests: Current status and future challenges. Environmental Pollution, 116, 363–372. 11822714 10.1016/s0269-7491(01)00212-3

[ece33089-bib-0003] Cairns, M. A. , Brown, S. , Helmer, E. H. , & Baumgardner, G. A. (1997). Root biomass allocation in the world's upland forests. Oecologia, 111, 1–11. 28307494 10.1007/s004420050201

[ece33089-bib-0004] Cambui, C. A. , Svennerstam, H. , Gruffman, L. , Nordin, A. , Ganeteg, U. , & Nasholm, T. (2011). Patterns of plant biomass partitioning depend on nitrogen source. PLoS ONE, 6, e19211. 21544211 10.1371/journal.pone.0019211 PMC3081341

[ece33089-bib-0005] Chapin, F. S. (1980). The mineral nutrition of wild plants. Annual Review of Ecology and Systematics, 11, 233–260.

[ece33089-bib-0006] Cheng, D. , & Niklas, K. (2007). Above‐ and belowground biomass relationships across 1543 forested communities. Annals of Botany, 99, 95–102. 17085476 10.1093/aob/mcl206 PMC2802968

[ece33089-bib-0007] Dixon, R. K. , Brown, S. , Houghton, R. A. , Solomon, A. M. , Trexler, M. C. , & Wisniewski, J. (1994). Carbon pools and flux of global forest ecosystems. Science, 263, 185–190. 17839174 10.1126/science.263.5144.185

[ece33089-bib-0008] Enquist, B. J. , & Niklas, K. J. (2001). Invariant scaling relations across tree‐dominated communities. Nature, 410, 655–660. 11287945 10.1038/35070500

[ece33089-bib-0009] Enquist, B. J. , & Niklas, K. J. (2002). Global allocation rules for patterns of biomass partitioning in seed plants. Science, 295, 1517–1520. 11859193 10.1126/science.1066360

[ece33089-bib-0010] Fang, J. Y. , Liu, G. H. , & Xu, S. L. (1996). Biomass and net production of forest vegetation in China. Acta Ecologica Sinica, 16(5), 497–508.

[ece33089-bib-0011] Friedlingstein, P. , Joel, G. , Field, C. B. , & Fung, I. Y. (1999). Toward an allocation scheme for global terrestrial carbon models. Global Change Biology, 5, 755–770.

[ece33089-bib-0012] Gholz, H. L. , Vogel, S. A. , Cropper, W. P. , Mckelvey, K. , Ewel, K. C. , Teskey, R. O. , & Curran, P. J. (1991). Dynamics of canopy structure and light interception in *Pinuselliottii* stands, North Florida. Ecological Monographs, 61, 33–51.

[ece33089-bib-0013] Hawkins, B. J. , Kiiskila, S. B. R. , & Henry, G. (1999). Biomass and nutrient allocation in Douglas fir and amabilis fir seedlings: Influence of growth rate and temperature. Tree Physiology, 19, 59–63. 12651333 10.1093/treephys/19.1.59

[ece33089-bib-0014] Hui, D. , Wang, J. , Le, X. , Shen, W. , & Ren, H. (2012). Influences of biotic and abiotic factors on the relationship between tree productivity and biomass in China. Forest Ecology Management, 264, 72–80.

[ece33089-bib-0015] Hui, D. , Wang, J. , Shen, W. , Le, X. , Ganter, P. , & Hai, R. (2014). Near isometric biomass partitioning in forest ecosystems of China. PLoS ONE, 9, e86550 https://doi.org/10.1371/journal pone.0086550 24466149 10.1371/journal.pone.0086550 PMC3899269

[ece33089-bib-0016] Huxley, J. S. , & Tessier, G. (1936). Terminology of relative growth. Nature, 137, 780–781.

[ece33089-bib-0017] Kang, M. Y. , Dai, C. , Ji, W. Y. , Jiang, Y. , Yuan, Z. Y. , & Chen, H. Y. H. (2013). Biomass and its allocation in relation to temperature, precipitation, and soil nutrients in Inner Mongolia grasslands, China. PLoS ONE, 8, e69561 https://doi.org/10.1371/journal.pone.0069561 23936045 10.1371/journal.pone.0069561 PMC3723834

[ece33089-bib-0018] Kellomaki, S. , & Wang, K. Y. (1996). Photosynthetic responses to needle water potentials in Scots pine after a four‐year exposure to elevated CO_2_ and temperature. Tree Physiology, 16, 765–772. 14871683 10.1093/treephys/16.9.765

[ece33089-bib-0101] King, J.‐S. , Giardina, C. P. , Pregitzer, K. S. , & Friend, A. L. (2007). Biomass partitioning in red pine (*Pinus resinosa*) along a chronosequence in the Upper Peninsula of Michigan. Canadian Journal of Forest Research, 37, 93–102.

[ece33089-bib-0019] Leuschner, C. , Moser, G. , Bertsch, C. , Roderstein, M. , & Hertel, D. (2007). Large altitudinal increase in tree root/shoot ratio in tropical mountain forests of Ecuador. Basic and Applied Ecology, 8, 219–230.

[ece33089-bib-0020] Li, H. T. , Han, X. G. , & Wu, J. G. (2005). Lack of evidence of 3/4 scaling of metabolism in terrestrial plants. Journal of Integrated Plant Biology, 47, 1173–1183.

[ece33089-bib-0021] Li, Z. , Kurz, W. A. , Apps, M. J. , & Beukema, S. J. (2003). Belowground biomass dynamics in the carbon budget model of the Canadian forest sector: Recent improvements and implications for the estimation of NPP and NEP. Canadian Journal of Forest Research, 33, 126–136.

[ece33089-bib-0022] Li, L. H. , Wang, Y. P. , Yu, Q. , Pak, B. , Eamus, D. , Yan, J. H. , … Baker, I. T . (2012). Improving the responses of the Australian community land surface model (CABLE) to seasonal drought. Journal of Geophysical Research‐Biogeosciences, 117(G4), 2156–2202.

[ece33089-bib-0023] Luo, T. X. , Brown, S. , Pan, Y. , Shi, P. , Ouyang, H. , Yu, Z. L. , & Zhu, H. Z. (2005). Root biomass along subtropical to alpine gradients: Global implication from Tibetan transect studies. Forest Ecology and Management, 206, 349–363.

[ece33089-bib-0024] Luo, Y. , Wang, X. , Zhang, X. , Booth, T. H. , & Lu, F. (2012). Root:shoot ratios across China's forests: Forest type and climatic effects. Forest Ecology and Management, 269, 19–25.

[ece33089-bib-0025] Luo, Y. J. , Wang, X. K. , Zhang, X. Q. , Ren, Y. , & Poorter, H. (2013). Variation in biomass expansion factors for China's forests in relation to forest type, climate, and stand development. Annals of Forest Science, 70, 589–599.

[ece33089-bib-0026] Matsui, Y. , Fukuda, Y. , Inoue, T. , & Matsushita, T. (2003). Effect of natural organic matter on powdered activated carbon adsorption of trace contaminants: Characteristics and mechanism of competitive adsorption. Water Research, 37, 4413–4424. 14511712 10.1016/S0043-1354(03)00423-8

[ece33089-bib-0027] McConnaughay, K. D. M. , & Coleman, J. S. (1999). Biomass allocation in plants: Ontogeny or optimality? a test along three resource gradients. Ecology, 80, 2581–2593.

[ece33089-bib-0028] Mokany, K. , Raison, R. J. , & Prokushkin, A. S. (2006). Critical analysis of root:shoot ratios in terrestrial biomes. Global Change Biology, 12, 84–96.

[ece33089-bib-0029] Monk, C. (1966). Ecological importance of root/shoot ratios. Bulletin of the Torrey Botanical Club, 93, 402ff.

[ece33089-bib-0030] Mooney, H. A. , Kuppers, M. , Koch, G. , Gorham, J. , Chu, C. , & Winner, W. E. (1988). Compensating effects to growth of carbon partitioning changes in response to SO_2_‐induced photosynthetic reduction in radish. Oecologia, 75, 502–506. 28312422 10.1007/BF00776411

[ece33089-bib-0031] Niklas, K. J. (2005). Modelling below‐ and aboveground biomass for non‐woody and woody plants. Annals of Botany, 95, 315–321. 15546927 10.1093/aob/mci028 PMC4246831

[ece33089-bib-0032] Niklas, K. J. (2006). A phyletic perspective on the allometry of plant biomass partitioning patterns and functionally equivalent organ categories. New Phytologist, 171, 27–40. 16771980 10.1111/j.1469-8137.2006.01760.x

[ece33089-bib-0033] Niklas, K. J. , & Enquist, B. J. (2001). Invariant scaling relationships for interspecific plant biomass production rates and body size. Proceedings of the National Academy of Sciences of the United States of America, 98, 2922–2927. 11226342 10.1073/pnas.041590298 PMC30241

[ece33089-bib-0034] Niklas, K. J. , & Enquist, B. J. (2002). On the vegetative biomass partitioning of seed plant leaves, stems, and roots. American Naturalist, 159, 482–497. 10.1086/339459 18707431

[ece33089-bib-0035] Peng, S. L. , & Zhang, Z. P. (1994). Biomass, productivity and light use efficiency of the zonal vegetation in Dinghushan Nature Reserve. Science in China (Series B), 24(5), 497–502.

[ece33089-bib-0036] Poorter, H. , & Nagel, O. (2000). The role of biomass allocation in the growth response of plants to different levels of light, CO_2_, nutrients and water: A quantitative review. Australian Journal of Plant Physiology, 27, 595–607.

[ece33089-bib-0037] Read, J. J. , & Morgan, J. A. (1996). Growth and partitioning in *Pascopyrumsmithii* (C‐3) and *Boutelouagracilis* (C‐4) as influenced by carbon dioxide and temperature. Annals of Botany, 77, 487–496.

[ece33089-bib-0038] Reich, P. B. (2002). Root–shoot relations: Optimality in acclimation and adaptation or the “Emperor's New Clothes”? In Waisel Y., Eshel A., & Kafkafi U. (Eds.), Plant Roots: The Hidden Half, 3rd ed. (pp. 205–220). Basel, Switzerland: Marcel Dekker.

[ece33089-bib-0039] Reich, P. B. , et al. (2014). Temperature drives global patterns in forest biomass distribution in leaves, stems, and roots. Proceedings of the National Academy of Sciences of the United States of America, 111, 13721–13727. 25225412 10.1073/pnas.1216053111 PMC4183289

[ece33089-bib-0102] Sheng, W.‐T. , & Fan, S.‐H. (2005). Long‐term productivity of Chinese fir plantations. Beijing: Science Press, 251 pp.

[ece33089-bib-0040] Smith, R. J. (1980). Rethinking Allometry. Journal of Theoretical Biology, 87, 97–111. 7206755 10.1016/0022-5193(80)90222-2

[ece33089-bib-0041] Vogel, J. G. , Bond‐Lamberty, B. P. , Schuur, E. A. G. , Gower, S. T. , Mack, M. C. , O'Connell, K. E. B. , … Ruess, R. W. (2008). Carbon allocation in boreal black spruce forests across regions varying in soil temperature and precipitation. Global Change Biology, 14, 1503–1516.

[ece33089-bib-0042] Wang, X. P. , Fang, J. Y. , & Zhu, B. (2008). Forest biomass and root‐shoot allocation in northeast China. Forest Ecology and Management, 255, 4007–4020.

[ece33089-bib-0043] Wang, L. , Li, L. , Chen, X. , Tian, X. , Wang, X. , & Luo, G. (2014). Biomass Allocation Patterns across China's Terrestrial Biomes. PLoS ONE, 9(4), e93566 https://doi.org/10.1371/journal.pone.0093566. 24710503 10.1371/journal.pone.0093566 PMC3977935

[ece33089-bib-0044] West, G. B. , Brown, J. H. , & Enquist, B. J. (1997). A general model for the origin of allometric scaling laws in biology. Science, 276, 122–126. 9082983 10.1126/science.276.5309.122

[ece33089-bib-0045] West, G. B. , Enquist, B. J. , & Brown, J. H. (2009). A general quantitative theory of forest structure and dynamics. Proceedings of the National Academy of Sciences of the United States of America, 106, 7040–7045. 19363160 10.1073/pnas.0812294106 PMC2678466

[ece33089-bib-0046] Yang, Y. H. , Fang, J. Y. , Ji, C. J. , & Han, W. X. (2009). Above‐ and belowground biomass allocation in Tibetan grasslands. Journal of Vegetation Science, 20, 177–184.

[ece33089-bib-0047] Zhang, H. , Song, T. Q. , Wang, K. L. , Wang, G. X. , Liao, J. X. , Xu, G. H. , & Zeng, F. P. (2015). Biogeographical patterns of biomass allocation vary with climate, soil and forest characteristics in China. Environmental Research Letters, 10, 044014.

[ece33089-bib-0048] Zhang, H. , Wang, K. L. , Xu, X. L. , Song, T. Q. , Xu, Y. F. , & Zeng, F. P. (2015). Biogeographical patterns of biomass allocation in leaves, stems and roots in China's forests. Scientific Reports, 5, 15997; https://doi.org/10.1038/srep15997 26525117 10.1038/srep15997 PMC4630587

